# Protein Supplementation Increases Adaptations to Low-Volume, Intra-Session Concurrent Training in Untrained Healthy Adults: A Double-Blind, Placebo-Controlled, Randomized Trial

**DOI:** 10.3390/nu16162713

**Published:** 2024-08-15

**Authors:** Dejan Reljic, Nilas Zieseniss, Hans Joachim Herrmann, Markus Friedrich Neurath, Yurdagül Zopf

**Affiliations:** 1 Department of Medicine 1, University Hospital Erlangen, Friedrich-Alexander University Erlangen-Nürnberg, 91054 Erlangen, Germany; nilas.zieseniss@gmail.com (N.Z.); hans.herrmann@uk-erlangen.de (H.J.H.); markus.neurath@uk-erlangen.de (M.F.N.); yurdaguel.zopf@uk-erlangen.de (Y.Z.); 2 Hector-Center for Nutrition, Exercise and Sports, Department of Medicine 1, University Hospital Erlangen, Friedrich-Alexander University Erlangen-Nürnberg, 91054 Erlangen, Germany; 3 German Center Immunotherapy (DZI), University Hospital Erlangen, Friedrich-Alexander University Erlangen-Nürnberg, 91054 Erlangen, Germany

**Keywords:** low-volume exercise, HIIT, resistance training, interference effect, whey protein

## Abstract

Combined endurance and resistance training, also known as “concurrent training”, is a common practice in exercise routines. While concurrent training offers the benefit of targeting both cardiovascular and muscular fitness, it imposes greater physiological demands on the body compared to performing each modality in isolation. Increased protein consumption has been suggested to support adaptations to concurrent training. However, the impact of protein supplementation on responses to low-volume concurrent training is still unclear. Forty-four untrained, healthy individuals (27 ± 6 years) performed two sessions/week of low-volume high-intensity interval training on cycle ergometers followed by five machine-based resistance training exercises for 8 weeks. Volunteers randomly received (double-blinded) 40 g of whey-based protein (PRO group) or an isocaloric placebo (maltodextrin, PLA group) after each session. Maximal oxygen consumption (VO_2max_) and overall fitness scores (computed from volunteers’ VO_2max_ and one-repetition maximum scores, 1-RM) significantly increased in both groups. The PRO group showed significantly improved 1-RM in all major muscle groups, while the PLA group only improved 1-RM in chest and upper back muscles. Improvements in 1-RM in leg muscles were significantly greater in the PRO group versus the PLA group. In conclusion, our results indicate that adaptations to low-volume concurrent training, particularly leg muscle strength, can be improved with targeted post-exercise protein supplementation in untrained healthy individuals.

## 1. Introduction

Adequate levels of cardiorespiratory [1,2,3,4] and muscular fitness [1,5,6] are crucial determinants for the maintenance of general health and for preventing numerous chronic diseases. It has been documented, for example, that the degree of maximal oxygen consumption (VO_2max_) as an indicator of cardiorespiratory fitness is a key predictor of cardiovascular disease and overall mortality, even stronger than traditional risk factors, such as obesity, hypertension, type 2 diabetes mellitus, or nicotine abuse [7,8]. Additionally, research indicates that muscle strength is an independent and significant factor related to morbidity and mortality [6,9]. Thus, guidelines [10,11] advocate that individuals should participate in both regular aerobic and muscle-strengthening activities to maintain/improve cardiorespiratory as well as muscular fitness. Accordingly, prescriptions for structured exercise programs typically involve combined endurance and resistance training—also termed “concurrent training”—to promote holistic fitness and health benefits.

Despite the well-accepted additive benefits of combined exercise programs with regard to overall fitness and health outcomes, research has highlighted that concurrent training—particularly when both modalities are carried out consecutively in the same session (commonly referred to as “intra-session concurrent training”)—can lead to greater physiological stress by challenging multiple systems (cardiovascular, muscular) simultaneously [12,13]. It has been suggested that previous endurance training may compromise subsequent resistance exercise quality and vice versa, due to residual fatigue and/or reduced substrate availability (e.g., depleted glycogen levels resulting in increased skeletal muscle protein breakdown) [14,15,16]. Moreover, there is a body of evidence suggesting that adaptations to endurance and resistance training may interfere with each other under certain circumstances [14,15,17,18,19]. This so-called interference effect was first observed in a pioneering study by Hickson [20], who found that simultaneous endurance and resistance training resulted in a reduced capacity to develop muscle strength when compared to resistance training alone in recreationally active subjects. Although this finding was not always confirmed in follow-up studies, multiple investigations showed similar results, indicating that particularly muscle strength development and hypertrophy can potentially be diminished by concurrent training [14,15,17,18,19], most likely due to antagonistic molecular mechanisms underlying adaptations to both types of exercise [14,15,17]. Additionally, it has been demonstrated that untrained individuals can experience lower VO_2max_ improvements with concurrent versus endurance training only [21].

Practical recommendations to balance the increased physiological demands or mitigate potential interference effects of intra-session concurrent training are related to training variables (e.g., type of exercise, volume, and intensity) [19] and nutritional strategies [22]. Regarding nutrition, protein supplementation has been particularly highlighted in recent systematic reviews as a potential approach to optimize synthesis of muscle protein, aid muscle repair and growth, and support strength adaptations during concurrent training [22,23,24,25]. There is evidence, for example, that ingesting 20–40 g of protein immediately after an exercise session can provide a beneficial impact on muscle protein synthesis and performance responses to concurrent training [24]. Furthermore, it has been reported that post-exercise protein intake may contribute to improvements in cardiovascular fitness by supporting, for example, the forming of new capillaries, oxygen-transporting proteins, and mitochondrial proteins [26]. However, in this context, it must be pointed out that the majority of previous trials investigating the effects of protein supplementation or increased dietary protein intake on concurrent training adaptations involved athletes or trained/physically active individuals [27,28,29,30,31,32,33,34,35,36,37,38,39,40,41,42,43,44,45,46,47] and used higher-volume exercise programs, such as prolonged continuous endurance training or longer-duration interval training protocols combined with multiple-set resistance training regimens [27,28,29,30,31,32,33,35,37,38,39,40,42,43,44,45,46,47,48,49,50,51,52].

Currently, only a small number of investigations [28,48,49,50,51,52,53] have been conducted with sedentary/untrained samples, of which only one trial examined the influence of protein supplements on changes in cardiorespiratory fitness compared to concurrent training without supplementation [51]. In that study, Lockwood et al. [51] found that absolute VO_2max_ only improved in conjunction with whey protein supplementation over a period of 10 weeks of concurrent endurance and resistance exercise in a group of sedentary, overweight females and males. Given that VO_2max_ improvements following concurrent training, in comparison to isolated endurance exercise, were found to be particularly blunted in untrained individuals [21], there is clearly a need for more research to investigate whether protein supplementation can improve adaptations to concurrent endurance and resistance training in novice exercisers. Moreover, to our knowledge, it has not yet been investigated whether individuals engaged in more time-efficient, “low-volume” training programs may also benefit from targeted protein intake after completion of the exercise session. Low-volume training types, including low-volume high-intensity interval training (LOW-HIIT) [54,55], a specific form of interval endurance exercise (involving, by definition, ≤10 min of intensive exercise during a training session of ≤30 min duration, including periods of warm-up and cool-down [55]) and low-volume resistance training (LOW-RT, previously defined as <12 weekly exercise sets per muscle group [56]), have gained increasing popularity among exercisers who have tight time schedules and thus have become a fruitful topic of research in recent years [57,58].

The aim of this study was therefore to address these research gaps by examining the effects of protein supplementation (40 g of whey-based protein) post-exercise on adaptations of VO_2max_, muscle strength and body composition after an 8-week low-volume concurrent training program comprising LOW-HIIT and LOW-RT (two sessions per week), in previously sedentary, healthy men and women. We hypothesized that both groups would show improve physical fitness indices, but that post-session protein supplementation would increase the concurrent training-induced responses of cardiorespiratory and muscular fitness compared to an isocaloric placebo.

## 2. Materials and Methods

### 2.1. Design of the Study

This investigation was a randomized, placebo-controlled, double-blind trial involving a concurrent training intervention (LOW-HIIT followed by LOW-RT) of 8 weeks duration with two arms (experimental group and placebo control group). The experimental condition consisted of 2 weekly sessions of concurrent LOW-HIIT and LOW-RT plus post-exercise whey-based protein supplementation (PRO group). The control condition consisted of the same concurrent training program plus post-exercise supplementation of an isocaloric placebo (PLA group). Primary outcomes of the study were VO_2max_, maximum strength values, defined as one-repetition maximum (1-RM) for the five main muscle groups (chest, upper back, abdominals, lower back, and legs), and overall fitness (Fit score, computed as the mean of VO_2max_ and the average 1-RM value of the five muscle groups). Secondary outcomes were body composition parameters, described in more detail in Section 2.3.3.

**Figure 1:**
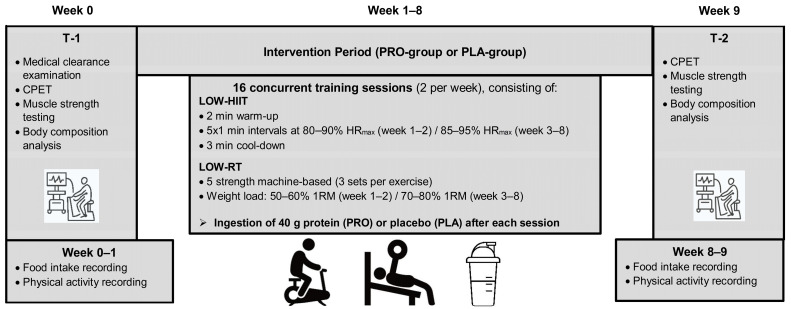
Study timeline. T-1 = pre-intervention; T-2 = post-intervention; LOW-HIIT = low-volume high-intensity interval training; LOW-RT = low-volume resistance training; CPET = cardiopulmonary exercise testing.

The outcome measurements were conducted 1 week preceding the onset of the exercise program (i.e., week 0, T-1) and in the first week after completion of the exercise program (i.e., week 9, T-2). The timeline of the trial is illustrated in Figure 1. After T-1, volunteers were allocated to the two groups by stratified-randomization based on their baseline VO_2max_ (<35 mL/kg/min, or ≥35 mL/kg/min), age (<30 years, or ≥30 years), and sex (male or female) using the software MinimPy (GNU General Public License version 3.0 [59]). Randomization was conducted by a researcher not engaged in the collection of data. All volunteers in the trial were fully briefed on the study’s scope, which complied with the Declaration of Helsinki, and signed an informed consent prior to study inclusion. The study was authorized by the Medical Faculty Ethics Committee of Friedrich–Alexander University Erlangen—Nürnberg (approval 147_19B) and registered at ClinicalTrials.gov (ID NCT04359342).

### 2.2. Study Volunteers

Participant recruitment involved advertising in local newspapers and social media platforms. Interested persons contacted study staff via email or by telephone to determine if they were eligible to participate. Eligibility criteria for the study included that volunteers were at least 18 years of age, led a mostly sedentary lifestyle as defined elsewhere [60], and were not participating in any other exercise or nutrition intervention. Exclusion criteria included pregnancy, clinical diagnosis of coronary disease, oncological disease, major orthopedic disorders, or other serious health problems that would rule out safe involvement in physical exercise. All volunteers consented to maintaining their current lifestyle habits during the study to minimize possible bias. We based our sample size calculation on results of a recent meta-analysis [24], which demonstrated a large pooled effect size (d  =  0.89) of the impact of protein supplementation on improvements in performance outcomes in response to concurrent training. Accordingly, an a priori estimation of sample size, expecting a large effect size for repeated-measure ANOVA (f = 0.45), indicated that a total of 20 participants (N = 10 for each group) would be adequate to yield a power of 95% with a significance level of 5% (G*Power, version 3.1.9.2). In order to take possible dropouts into account, the aim was to recruit 20 participants per group.

### 2.3. Outcome Measurements

The baseline testing procedures (T-1) were conducted 1 week prior to the onset of the exercise program. The post-testing (T-2) took place within the first week after the completion of the 8-week exercise program, with at least 3 days between the last exercise session in order to ensure sufficient recovery. T-1 and T-2 were scheduled at a similar time of day to reduce potential circadian influences. Additionally, care was taken that both testing days were performed within the same menstrual cycle in all female volunteers. Volunteers were advised to report to the laboratory in an overnight-fasted state, to abstain from alcohol, and avoid vigorous physical activity for a minimum period of 24 h prior to their visit. Measurements were strictly standardized as specified below and performed in stable ambient conditions (22–24 °C, and 30–50% air humidity). At T-1, study outcome measurements were preceded by a medical clearance examination, including medical history recording, blood pressure measurements, 12-lead resting electrocardiography, and evaluation of standard blood and urine laboratory values to assure the safety of participation in the training program. All measurements and examinations were executed investigator-blinded, meaning that personnel collecting the data had no knowledge of volunteers’ group assignment.

#### 2.3.1. Body Composition Measurements

Upon arrival at the laboratory, volunteers were requested to void their bladder, and afterwards, to remain in a seated position for 5 min. Subsequently, multifrequency segmental bioelectrical impedance analysis was performed using a validated analyzer (seca mBCA 515, Seca, Hamburg, Germany) [61] to assess the body weight, body fat mass, skeletal muscle mass, and total body water of the volunteers. Furthermore, volunteers’ waist circumference was obtained in the standing position to the closest millimeter. Measurements were performed approximately midway between the lower edge of the last palpable rib and the upper iliac crest along the mid-axillary line using a flexible tape.

#### 2.3.2. Cardiopulmonary Exercise Test (CPET)

CPETs were carried out using a stationary electronically braked cycle ergometer (Corival cpet, Lode, Groningen, The Netherlands) to assess VO_2max_, maximal power output (W_max_), and maximal heart rate (HR_max_). Additionally, volunteers’ power output at the ventilatory threshold (W_VT_) was assessed by means of the V-slope method (i.e., plot of carbon dioxide release versus oxygen consumption) to determine submaximal endurance capacity. Following a 1 min adaptation, CPET commenced at 50 W, with the power output progressively increasing by 12.5 W/min (females) and 15 W/min (males) until reaching voluntary exhaustion. Exhaustion was reached within 8–12 min in most volunteers, as per suggested guidelines for exercise testing [60]. Heart rate was recorded constantly with a 12-lead ECG device (custo cardio 110, custo med, Ottobrunn, Germany). Oxygen consumption and carbon dioxide release were obtained constantly with a breath-by-breath, open-circuit metabolic cart (Metalyzer 3B-R3, Cortex Biophysik, Leipzig, Germany). Oxygen consumption and carbon dioxide release data were averaged every 10 s. To verify that maximal exertion had been achieved, volunteers had to meet a minimum two of the specified criteria: plateauing of oxygen consumption, a respiratory exchange ratio of ≥1.1, an age-related HR_max_ of ≥90% (computed according to the formula 220—age), and a rate of perceived exertion of ≥19 on the Borg scale [62], as recommended elsewhere [63]. CPET data were used to set the volunteers’ personalized LOW-HIIT heart rate zones.

#### 2.3.3. Determination of One-Repetition Maximum Strength and Overall Fitness Z Score

After a brief familiarization with test procedures and local warm-up of the target muscles, volunteers conducted a modified 1-RM test of the following muscles: chest, upper back, abdominals, lower back, and legs. While a “classical” 1-RM test typically aims to determine the maximal weight load that can be lifted for one complete repetition, the modified 1-RM test utilized in the present trial involved performing multiple repetitions to predict 1-RM. This method is considered to have a lower a lower risk of injury and is therefore advocated for untrained collectives [64]. The tests were supervised by certified physiotherapists or sports therapists on five machines in the following standardized order: chest press, lat pulldown machine, lower back machine, abdominal crunch, and leg press (TechnoGym, Neu-Isenburg, Germany). On each machine, volunteers were required to raise the applied weight until reaching muscular failure. As recommended elsewhere [65], the number of repetitions was not to exceed six to ensure accurate 1-RM predictions. If more than six repetitions were completed, the weight was increased and a following attempt was executed after a 3 min recovery. The load that could be lifted for six repetitions was usually determined within three tries. Afterwards, 1-RM values were estimated based on the following formula [66]:

1-RM = 100 × load rep/(102.78 × 2.78 × rep)

The test results were utilized to determine volunteers’ weight load for the resistance training exercises as specified below (2.5). At the beginning of training week 4, 1-RM tests were repeated to account for progression and to reestablish the respective weight loads. Furthermore, an overall fitness (Fit score) was computed at T-1 and T-2 as the mean value of each fitness sub-component (cardiorespiratory and muscular fitness) as follows:

Fit score = (VO_2max_ + average 1-RM from the five muscle groups)/2

### 2.4. Daily Nutrition and Physical Activity Monitoring

Volunteers were instructed to record their dietary intake on three days in a row during the week prior to the onset of the exercise program and during the last training week with the help of a standardized 24 h food protocol (Freiburger Ernährungsprotokoll; Nutri-Science, Freiburg, Germany). A registered dietitian analyzed all dietary records using software (PRODI 6 expert, Nutri-Science, Freiburg, Germany). Furthermore, volunteers recorded their habitual physical activities on a daily basis in an activity diary. All recorded physical activities were categorized based on metabolic equivalents (METs), according to Ainsworth et al. [67]: light (<3 METs), moderate (3–6 METs), or vigorous (>6 METs). The average MET score over 24 h was used to assess the daily physical activity level (PAL).

Based on the individual PAL and anthropometric values, volunteers received personalized dietary advice to keep a consistent nutritional intake throughout the intervention period. The dietary advice adhered to guidelines from the German Nutrition Society [68,69]. Volunteers’ resting metabolic expenditure (REE) was calculated by the following established equations [70]:

Men: REE (kcal/day) = 66.5 + 13.8 × weight (kg) + 5.0 × size (cm) − 6.8 × age (years)

Women: REE (kcal/day) = 655 + 9.6 × weight (kg) + 1.8 × size (cm) − 4.7 × age (years)

Caloric requirements per day were computed by multiplication of REE with PAL values. Volunteers were instructed to ingest 10–15% of daily energy from protein, 30–35% from fat, and ≥50% from carbohydrates [68]. Handouts containing meal planning advice and detailed instructions were provided to help volunteers implement the dietary recommendations at home.

### 2.5. Concurrent Training Program

During the 8-week intervention period, volunteers conducted two weekly concurrent training sessions for a total of sixteen sessions. To maximize compliance, volunteers could schedule all exercise sessions on an individual basis throughout the opening hours of the Training Center, with at least 1 day’s rest in between to ensure proper recovery. The exercise sessions consisted of sequential LOW-HIIT and LOW-RT, which were all supervised by certified sports therapists or physiotherapists.

All sessions commenced with a LOW-HIIT cycle ergometer protocol, which was adapted from Reljic et al. [71]. Briefly, volunteers warmed up with low-intensity cycling for 2 min. Subsequently, volunteers performed 5 intervals of 1 min duration at 80–90% of HR_max_ in week 1 and week 2. From week 3 on, training intensity was increased to a target heart rate range of 85–95% of HR_max_, to be achieved during the intervals. Throughout each session, volunteers wore a heart rate chest strap (acentas, Hörgertshausen, Germany) to measure their individual heart rate during exercise. Heart rate data were saved for later analysis using a software program (HR monitoring team system, acentas, Hörgertshausen, Germany). During each interval bout, volunteers were directed to adjust the cadence and/or the load resistance of the ergometer to achieve their pre-defined heart rate zones. Intervals were separated by a 1 min recovery period with low-intensity cycling. The last interval bout was followed by a 3 min cool-down period at a self-selected low-intensity pace. According to previous definitions of “low-volume HIIT” [55], the total duration of LOW-HIIT (warm-up and cool-down included) was 14 min/session.

After completing LOW-HIIT, volunteers performed five resistance exercises targeting the major muscle groups, including chest muscles, upper back muscles, abdominal muscles, lower back muscles, and leg muscles, on the following training devices: chest press, lat pulldown machine, lower back machine, abdominal crunch, and leg press (TechnoGym, Neu-Isenburg, Germany). Each exercise was performed with 3 sets according to the following pattern: 2 s of concentric, 2 s of eccentric muscle work until the volunteer reached muscle failure, and 2 min rest between each set. As recommended for novice exercisers [72], the initial weight load during weeks 1–2 was set at 50–60% of 1-RM to achieve ~15–20 repetitions per set to accustom volunteers to resistance training and thereafter progressed to 70–80% of 1-RM, targeting 8–12 repetitions per set. Previously defined as “low-volume resistance training” [56], the resistance training part of the exercise session involved only 6 sets per muscle group per week. Thus, total time per session, including both LOW-HIIT and LOW-RT, was ~57 min per session (~114 min exercise per week).

### 2.6. Supplementation

Following the conclusion of every exercise session, volunteers received (double-blinded) 40 g of a whey-based protein supplement (Fresubin Protein, Fresenius Kabi, PRO group) or an isocaloric placebo (maltodextrin) with the same taste (MaltoCal 19, MetaX, PLA group). According to previous research, consumption of 40 g of protein after termination of exercise appears to be a very effective approach to increase synthesis rates of muscle protein in healthy subjects [73]. Table 1 presents the calorie and macronutrient composition of each supplement. Both supplements were prepared with 150 mL of low-fat milk (46 kcal/100 mL, 3.4 g protein, 4.8 g carbohydrates, 1.5 g fat) and administered in the form of a shake in identical non-transparent drinking cups. Based on the medical history survey obtained at study entry, none of the volunteers reported being lactose-intolerant or experiencing any associated clinical symptoms after milk consumption. Supplement preparation and delivery were carried out by staff members who were not engaged in the collection and analysis of study outcomes. Once data collection was complete, the group allocation of each volunteer was revealed. Volunteers were requested to document their personal responses with the supplements, including any relevant observations pertaining to their taste and any adverse effects they may have experienced as a result of taking them (e.g., nausea, bloating, or stomach pain). Furthermore, to assess blinding success, volunteers were asked to estimate which supplement they thought they had received after completion of the intervention using the following answer options: “protein”, “placebo”. or “I do not know”.

### 2.7. Statistical Analysis

SPSS version 24.0 software (SPSS Inc., Chicago, IL, USA) was used for analyses. The data were first checked for whether they exhibited a normal distribution using the Shapiro–Wilk test. A 2 × 2 repeated-measure ANOVA was subsequently conducted to examine the data, with the objective of analyzing the main effects of group (PRO vs. PLA), time (T1 vs. T2), and their interaction. To assess whether sex influenced changes in the primary outcomes (VO_2max_, 1-RM-values, and Fit score), male and female sub-analyses were conducted. Levene’s test was utilized to confirm the homogeneity of variance. In instances where ANOVA revealed the existence of a significant main effect or interaction, Holm–Sidak post hoc tests were used for multiple and between-group comparisons and post hoc paired *t*-tests were applied to identify changes within groups [74,75]. In cases where the data exhibited a skewed distribution, logarithmic or square root transformations were applied, and the identical analyses described above were conducted on the transformed data. If normalization was not achieved through transformation (W_VT_, 1-RM chest press, 1-RM lat pulldown machine, Fit score and PAL values), Friedman two-way analysis of variance was employed. In cases of significant results, Dunn’s Bonferroni post hoc tests were used for comparisons between groups, and Wilcoxon and Mann–Whitney post hoc tests were carried out for comparisons within groups, respectively. Moreover, effect sizes were determined using partial eta-squared (*ήp*^2^) for ANOVAs and Kendall’s coefficient of concordance (*W*) for the Friedman tests. Based on the established literature [76], effect sizes were deemed small (≤0.01), medium (≥0.06) and large (≥0.14) for *ήp*^2^, and small (≤0,10), medium (≥0.30), and large (≥0.50) for *W*. In all statistical tests, the threshold of significance was defined at *p* < 0.05. Data are reported as means ± standard deviation (SD). Changes between T-1 and T-2 are presented with 95% confidence intervals (95% CI).

## 3. Results

### 3.1. Study Flow

**Figure 2:**
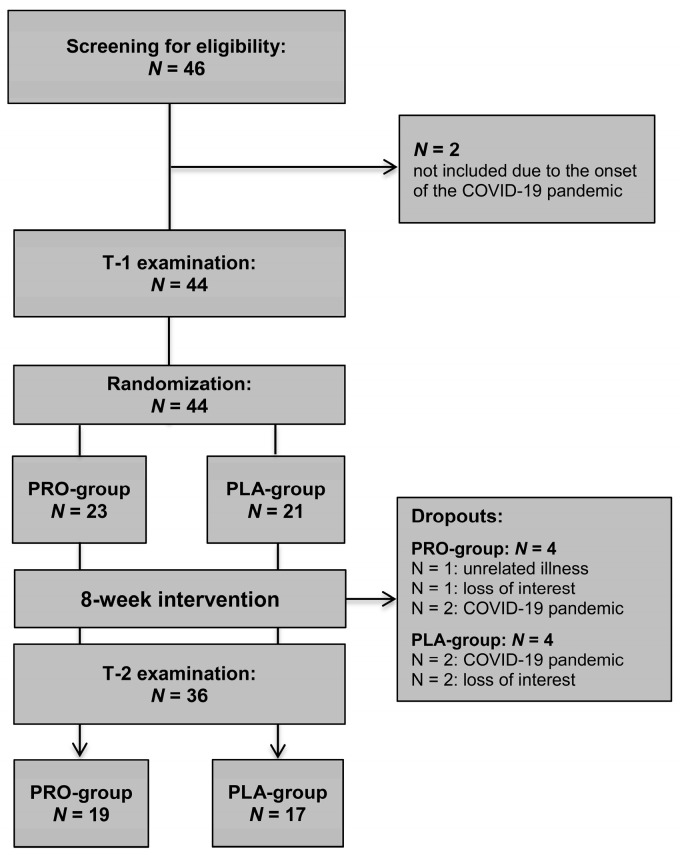
Study flowchart.

In total, 46 individuals were screened for eligibility, of whom 44 were included and randomly allocated to the PRO group (N = 23) or PLA group (N = 21). Two dropouts of eligible candidates occurred due to the onset of the COVID-19 pandemic. All participating volunteers were free of medications, except for two women (N = 1; each group), who were taking contraceptives. During the study, eight volunteers dropped out (PRO group, N = 4; 25% females, PLA group, N = 4; 100% females). The specific reasons for dropout are illustrated in Figure 2. Thus, the study concluded with a total of 36 volunteers having been analyzed (PRO group, N = 19, 63% females, 26 ± 4 years; PLA group, N = 17, 53% females, 27 ± 6 years).

### 3.2. Training Data, Adverse Events, and Volunteers’ Evaluations

There were no significant between-group differences in primary outcomes at T-1. Moreover, no notable sex-based differences were identified in the observed alterations in VO_2max_ and 1-RM values. Thus, the results for both sexes were combined in all analytical procedures. Training compliance (indicated by the percentage of scheduled sessions attended) was notably high (PRO group: 93% ± 10%, PLA group: 93% ± 8%). The mean peak heart rate reached during the intervals of the LOW-HIIT protocol corresponded to 95% ± 2% of volunteers’ HR_max_, indicating successful attainment of the targeted intensity of exercise. The mean heart rate throughout the whole LOW-HIIT protocol (warm-up, intervals, recovery between intervals, and cool-down calculated together) equaled 78% ± 3% of volunteers’ HR_max_. All volunteers managed to lift the prescribed weight loads, and completed 17 ± 2 repetitions per set during weeks 1–2 and 10 ± 1 repetitions per set during weeks 3–8.

Throughout the whole intervention period, no adverse events associated with the exercise program were documented. A mean score of 6.0 ± 0.7 was recorded on a 7-point rating scale, ranging from 1 (indicating that the exercise program was “not enjoyable at all”) to 7 (representing “extremely enjoyable”). This indicates that the training program was rated as highly enjoyable by the volunteers. Moreover, 90% of volunteers expressed an intention to continue with low-volume concurrent training after the study. In the PRO group, no complaints or intolerance were reported after consuming the protein supplement. In the PLA group, only a small number of minor adverse events were documented following consumption of the maltodextrin supplement, including mild gastric discomfort (N = 1), flatulence (N = 2), and mild nausea (N = 1). A 7-point Likert scale was employed to assess the palatability of the supplements, with an average rating of 5.0 ± 1.6 for the protein supplement and 5.5 ± 1.0 for the placebo. The majority of volunteers (N = 26, 76%) declared that they were unsure which supplement they received during the intervention. Five (15%) volunteers correctly identified which supplement they received (PRO group: N = 3, PLA group: N = 2). Three (9%, all PLA group) volunteers incorrectly identified the supplement they received.

### 3.3. Nutritional Intake and Daily Physical Activity

There were no significant differences in regular diet or physical activity habits within the groups or between them. Table 2 presents the dietary intake and physical activity data for each group, recorded both before the intervention period and during the final week of training.

### 3.4. Anthropometric Data

There were no significant main or interaction effects for any anthropometric parameter, except for a significant main effect of time in waist circumference (*p* = 0.033, *ή*^2^ = 0.13). Post hoc tests revealed a reduction in waist circumference by 2.0 cm (95% CI: –3.3 to –0.1 cm, *p* = 0.038) in the PRO group. Table 3 displays the data specific to each group at both T-1 and T-2.

### 3.5. Cardiorespiratory Fitness Data

**Figure 3:**
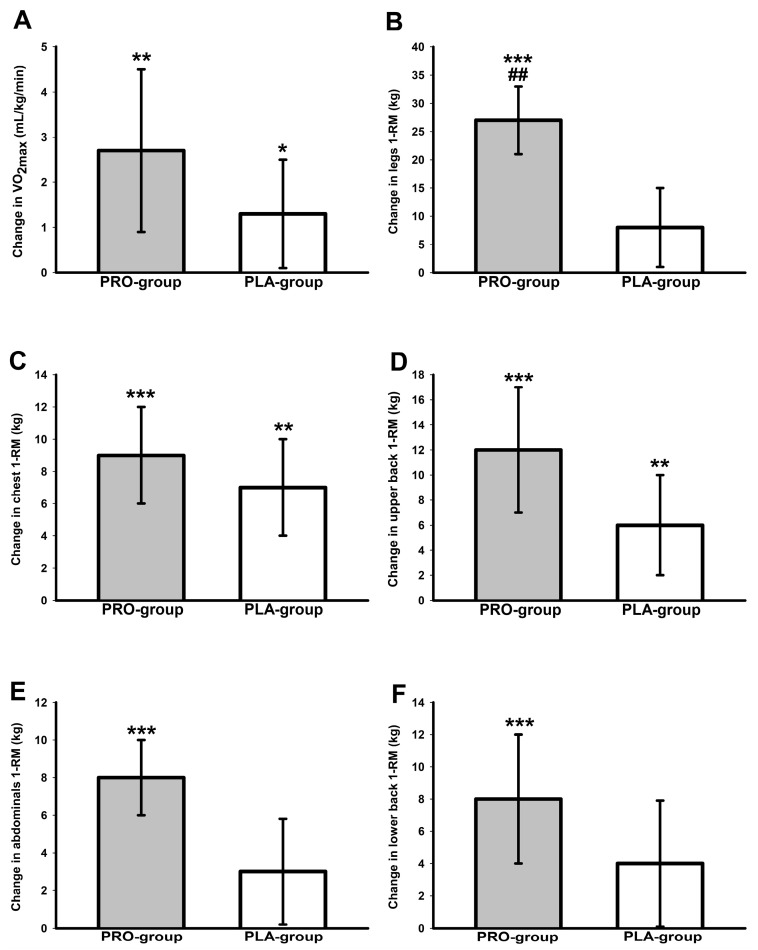
Changes in VO_2max_ (**A**), legs 1-RM (**B**) chest 1-RM (**C**), upper back 1-RM (**D**), abdominals 1-RM (**E**), and lower back 1-RM (**F**). * (*p* < 0.05), ** (*p* < 0.01), *** (*p* < 0.001): significant change between T-1 and T-2. ^##^ (*p* < 0.01): significant difference between PRO group and PLA group.

Significant main effects of time were found for relative VO_2max_ (*p* < 0.001, *ή*^2^ = 0.30) and absolute VO_2max_ (*p* < 0.001, *ή*^2^ = 0.28), as well as for relative W_max_ (*p* < 0.001, *ή*^2^ = 0.61) and absolute W_max_ (*p* < 0.001, *ή*^2^ = 0.66). Post hoc tests identified increases in absolute VO_2max_ (PRO group: 0.3 L/min, 95% CI: 0.1 to 0.4 L/min, *p* = 0.005; PLA group: 0.1 L/min, 95% CI: 0 to 0.2 L/min, *p* = 0.011), relative VO_2max_ (PRO group: 2.7 mL/kg/min, 95% CI: 0.9 to 4.5 mL/min, *p* = 0.003; PLA group: 1.4 mL/min, 95% CI: 0.1 to 2.6 mL/min, *p* = 0.032) (Figure 3), absolute W_max_ (PRO group: 22 W, 95% CI: 15 to 29, *p* < 0.001; PLA group: 17 W, 95% CI: 10 to 24, *p* < 0.001) and relative W_max_ (PRO group: 0.4 W/kg, 95% CI: 0.2 to 0.5 W/kg, *p* < 0.001; PLA group: 0.2 W/kg, 95% CI: 0.1 to 0.3 W/kg, *p* < 0.001) in both groups. Group-specific values of all cardiorespiratory fitness outcomes at T-1 and T-2 are presented in Table 4.

### 3.6. One-Repetition Maximum Strength Data

Main effects of time were significant for 1-RM of chest (*p* < 0.001, *W* = 0.86), upper back (*p* < 0.001, *W* = 0.69), abdominals (*p* < 0.001, *ή*^2^ = 0.46), lower back (*p* = 0.001, *ή*^2^ = 0.26) and legs (*p* < 0.005, *ή*^2^ = 0.52). There was a significant group-by-time interaction for 1-RM in leg muscles (*p* = 0.007, *ή*^2^ = 0.20). Additionally, a strong trend for a group-by-time interaction was noted for 1-RM of abdominal muscles (*p* = 0.05, *ή*^2^ = 0.11). Post hoc tests revealed that the PRO group showed significantly (*p* < 0.001) improved 1-RM in all tested muscle groups (Figure 3), while the PLA group only improved 1-RM significantly in chest (*p* = 0.001) and upper back muscles (*p* = 0.002). Improvements in 1-RM of leg muscles (15 kg, 95% CI: 4 to 25 kg *p* = 0.003) were larger in the PRO group in comparison to the PLA group (Figure 3). Group-specific 1-RM values are shown in Table 5.

### 3.7. Overall Fitness Z Score

**Figure 4:**
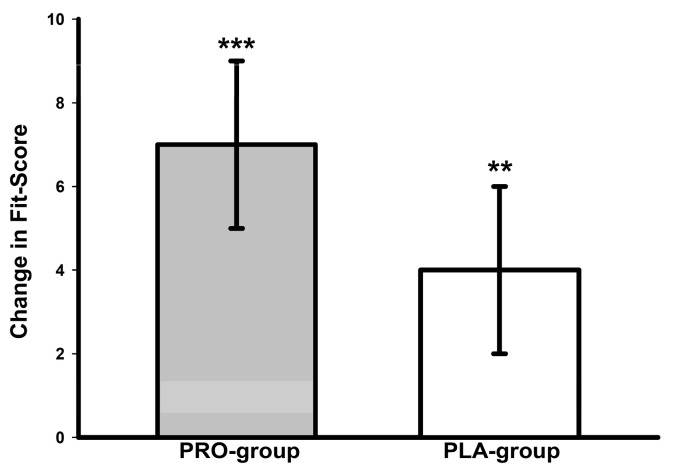
Changes in overall physical fitness Z score. ** *p* < 0.01, *** *p* < 0.001: significant change between T-1 and T-2.

A significant main time effect was detected for the Fit score (*p* < 0.001, *W* = 0.69). The Fit score significantly increased in both groups (PRO group, *p* < 0.001; PLA group, *p* = 0.002) (Figure 4). Group-specific Fit scores are presented in Table 5.

## 4. Discussion

To our knowledge, this trial is the first to evaluate the impact of a post-exercise protein supplementation following a low-volume concurrent training program consisting of combined LOW-HIIT and LOW-RT on physical fitness outcomes in untrained healthy individuals. Our findings provide important insights into the effectiveness of nutritional strategies in optimizing training adaptations in individuals performing low-volume concurrent cardiovascular and muscular training programs. The main results were as follows: (i) in accordance with our assumption, 8 weeks of low-volume concurrent training improved VO_2max_ and muscular strength in our examined cohort—irrespective of protein or placebo supplementation, (ii) improvements in leg muscle strength were significantly larger in the PRO group in comparison to the PLA group, pointing to a beneficial effect of post-exercise protein supplementation on lower body strength adaptations to combined intra-session LOW-HIIT and LOW-RT in previously untrained individuals.

The increase in VO_2max_ following the 8-week low-volume concurrent training program (~2.1 mL/kg/min, average of both groups) was in the range of the values observed in other investigations examining the impact of LOW-HIIT in healthy untrained or recreationally active individuals (1.2 to 7.2 mL/kg/min), including previous trials from our laboratory applying the identical LOW-HIIT protocol as in the current study [61,77,78,79,80,81,82,83]. Thus, in conjunction with previous findings, our data provide further evidence for the effectiveness of LOW-HIIT in improving cardiovascular health with relatively little time invested. Given the paramount importance of VO_2max_ for health and longevity [7,8], this finding has clinical significance and supports the role of LOW-HIIT in cardiometabolic disease prevention.

The observed improvements in 1-RM values (11–33%) in our study cohort are consistent with other research findings, suggesting that untrained individuals can experience notable increases in muscle strength following LOW-RT programs within a few weeks. A systematic review by Grgic et al. [84] found that LOW-RT can result in an average increase in 1-RM ranging from 20% to 35% over 6–12 weeks. A study by Schoenfeld et al. [85] reported that participants engaging in LOW-RT exhibited approximately a 25% increase in their 1-RM for both lower and upper body exercises after 8 weeks. Similarly, another study by Jenkins et al. [86] observed a 30% improvement in 1-RM values following a 12-week LOW-RT regimen in untrained young adults. These findings are important because increasing 1-RM is not only a measure of improved physical fitness but also a significant indicator of overall health. For instance, a study by Volaklis et al. [87] found that higher 1-RM is negatively correlated with the incidence of cardiovascular disease. Specifically, the study demonstrated that each standard deviation (SD) increase in muscle strength (equivalent to an approximate 15% increase in 1-RM) was associated with a 20–30% reduction in overall mortality risk and cardiovascular disease events. A meta-analysis [88] indicated that each 10% increase in muscle strength reduced the risk of type 2 diabetes by 12%. Evidence also suggests that higher muscle strength is associated with lower cancer mortality. Leong et al. [89] found that each 5 kg increase in handgrip strength was linked to a 17% decrease in cancer mortality. Moreover, the Health ABC study [90] reported that greater leg strength was associated with lower mortality rates in older adults. For every 10% increase in leg strength, there was an 11% reduction in the risk of death from all causes.

Taken together, our findings indicate that two weekly sessions of low-volume concurrent training, requiring less than 2 h of total time effort per week, can yield significant improvements in both cardiorespiratory and muscular fitness within only 8 weeks, which most likely translates into improved health status. However, when comparing the PRO group and the PLA group, it is a major finding of this study that the two groups noticeably differed regarding their improvements in leg muscle strength. This finding is of importance, since robust lower-extremity strength is not only essential for performing daily activities but also linked to several health outcomes, including the prevention and management of chronic diseases and a reduction in mortality risk [9,90,91,92]. For instance, it has been demonstrated that lower limb muscle strength is significantly associated with a lower risk of cardiometabolic disorders [91], cardiovascular disease [92] and all-cause mortality. Interestingly, it has been suggested that loss of strength in the lower limb muscles, in particular, significantly affects overall body functionality and may have a greater impact on mortality compared to upper limb muscle strength [9], which further underscores the relevance of our findings.

Regarding cardiorespiratory fitness, the improvement in VO_2max_ was ~1.3 mL/kg/min larger in the PRO group compared to the PLA group. Although this difference was not statistically significant, it can be deemed clinically meaningful [93], because it has been suggested that a 1 mL/kg/min improvement in VO_2max_ is related to a reduced risk of cardiovascular disease-related premature death by approximately 9% [94]. Moreover, it is of note that the PRO group showed substantial increases in 1-RM across all major muscle groups, while the PLA group showed improved 1-RM values only in the chest and upper back muscles. Although the changes in muscle strength (aside from leg muscle changes) were not statistically different between the two groups, these differences may be of clinical relevance [93]. Research suggests that small differences in 1-RM can be associated with important health benefits. For instance, in patients with chronic obstructive pulmonary disease (COPD), 1-RM improvements of approximately 5 kg in leg extension and 6 kg in chest press were identified as clinically significant. These improvements were correlated with better performance in functional tests such as the six-minute walk test [95]. In healthy populations, even small 1-RM differences, such as 1 kg, can translate to better performance in daily activities and reduce the risk of injury. It has been reported that minor gains in maximum strength can lead to improved balance, reduced fall risk, and better overall mobility, which are crucial for maintaining independence, especially in older adults [58,96]. Moreover, modest differences in muscle strength can have positive psychological effects, including enhanced self-esteem, reduced symptoms of depression and anxiety, and overall better mental health [96]. These findings collectively suggest that even small-to-modest differences in 1-RM values can be clinically relevant, contributing to better physical function, injury prevention, and mental health. Therefore, the observed differences in muscle strength changes between the PRO group and PLA group may be of clinical interest, even when the differences in most muscle groups did not reach statistical significance.

Consequently, our findings highlight the beneficial effects of post-exercise protein supplementation on leg strength adaptations, a critical variable linked to physical fitness and general health [9,90,91,92]. The observed increase in leg strength underscores the potential of protein supplementation to support lower-extremity muscle improvements and performance in response to low-volume concurrent training and aligns with previous research [24]. The absence of statistically significant differences in other measured physical fitness outcomes suggests that while protein supplementation may specifically benefit leg muscle strength, its effects on the adaptation of other muscle groups and cardiorespiratory fitness in response to low-volume concurrent training may need further investigation, potentially requiring larger samples and/or longer interventions. In this context, several hypothesized physiological mechanisms have been postulated to account for the beneficial influence of post-exercise protein supplementation to improve adaptations to concurrent training that primarily relate to muscle mass and strength adaptations [22,23,24,25]. First, protein intake after exercise stimulates synthesis of muscle protein by providing essential amino acids, which activate the mechanistic target of rapamycin (mTOR) pathway [97]. The mTOR activation is crucial for initiating the translation process necessary for muscle repair and growth. During concurrent training, the increased muscle protein synthesis response from protein supplementation can help offset the catabolic effects of previous endurance training, thereby promoting muscle hypertrophy and strength gains through subsequent resistance training [29]. Second, both endurance training (especially high-intensity training such as HIIT) and resistance training can cause exercise-induced muscle micro-trauma. Post-exercise protein supplementation provides the necessary substrates for muscle repair and promotes faster recovery [98]. This accelerated recovery allows for more effective subsequent training sessions, enhancing overall training adaptations. Additionally, post-exercise protein supplementation can positively influence the hormonal environment conducive to muscle anabolism. Protein ingestion has been shown to elevate the release of anabolic hormones such as insulin and growth hormone, which facilitate muscle protein synthesis and hypertrophy [99]. These impact on hormones can counteract the potential catabolic effects of endurance training, promoting a net anabolic state that supports resistance training adaptations. It is of note that neither of our two study groups experienced significant increases in skeletal muscle mass following the exercise program, suggesting that 8 weeks of LOW-RT may not be enough to induce substantial hypertrophic muscle changes, particularly when combined with additional LOW-HIIT within the same session. However, muscle strength increase results from both changes in muscle structure (in particular muscle hypertrophy) and neuronal adaptations, such as improved recruitment of motor units [100]. Although protein supplementation is primarily associated with beneficial effects on muscle hypertrophy, it has been reported that it may also play a role in neuronal adaptations by facilitating the repair and growth of neural tissues, ensuring optimal nerve function, and promoting the release of neurotransmitters involved in muscle contraction [101]. Moreover, it has been reported that protein supplementation can enhance muscle quality by promoting myofibrillar protein synthesis, thus increasing the density of contractile proteins within the muscle fibers and leading to more efficient force production per unit of muscle mass [102].

While the role of carbohydrates and fats in supporting endurance training adaptations is well established, emerging evidence suggests that protein availability and supplementation may also play a critical role. Changes in VO_2max_ are largely dependent on adaptations in cardiac output, stroke volume, capillary density, blood volume and mitochondrial capacity, which adapt at different rates in response to regular endurance training [103]. Protein intake supports repair and remodeling processes that are essential for cardiovascular and mitochondrial adaptations. Adequate protein availability, for example, is necessary to sustain the synthesis of new contractile proteins and enzymes that facilitate increased cardiac output and stroke volume [104]. In this regard, previous research has demonstrated that protein ingestion after endurance training can support formation of new capillaries and may enhance mitochondrial adaptations by promoting mitochondrial biogenesis and function [104,105,106].

There are some potential limitations of this study. First, we acknowledge that we did not rigorously monitor volunteers’ dietary intake or habitual activity patterns outside of the prescribed training sessions, except during the 3-day assessments at the beginning and end of the study. Also, volunteers were given general nutritional guidelines and recipes at the study’s onset, but their diets were not strictly standardized throughout the 8-week period. Thus, despite the absence of notable discrepancies in dietary intake and daily physical activities between the two observation periods or between the groups, it is not possible to entirely discount the potential influence of variations in habitual nutrition or physical activity on non-monitored days on the adaptations to the training program. Nonetheless, we note that our study aimed to determine if targeted protein supplementation post-exercise could enhance the adaptations to a low-volume concurrent exercise program without significantly altering volunteers’ habitual diets. Second, some conclusions are drawn from self-reported dietary intake and activity records. In this respect, it has been reported that people typically tend to underestimate their dietary intake and overestimate their engagement in physical activities, and that the act of recording itself may unconsciously alter behaviors [107]. However, we believe that the comprehensive guidance provided on the accurate recording of record dietary intake and daily activities likely minimized the potential for errors. Third, volunteers received a standardized dose of 40 g of protein following each training session. While it could be argued that matching the supplement dose to each volunteer’s body weight would have been more precise, it is not uncommon for post-exercise nutrient recommendations to be given as absolute values [108]. Further, previous studies using fixed protein supplementation have reported significant effects on myofibrillar muscle protein synthesis [73,109] and VO_2max_ [110,111], supporting the effectiveness of this approach. Fourth, volunteers’ body composition was determined with BIA. Although the utilized device has shown high accuracy in assessing skeletal muscle mass when compared to magnetic resonance imaging (MRI) and dual X-ray absorptiometry (DXA) (63), this method can have some limitations, including, for example, its sensitivity to hydration changes [112]. It is therefore possible that some pertinent distinctions in skeletal muscle adaptations between the PRO and PLA groups have been overlooked in this study. Fifth, one notable limitation of our study is the absence of biochemical markers, such as mTOR, which play a crucial role in muscle protein synthesis and hypertrophy. The inclusion of such markers would have allowed for a more comprehensive understanding of the molecular mechanisms driving the observed physiological changes. Future research should integrate biochemical analyses to provide deeper insight into the anabolic signaling pathways and their contribution to muscle strength and adaptation in response to exercise interventions. Finally, the 8-week duration of this study leaves questions about longer-term effects of protein supplementation following exercise on responses to low-volume concurrent training unanswered. Future research with extended intervention as well as with different training protocols (e.g., variations in the order of concurrent low-volume endurance and resistance training) is necessary to evaluate these questions. Despite these limitations, this study is the first double-blind, randomized, placebo-controlled investigation to evaluate the impact of targeted protein supplementation following a concurrent low-volume exercise program on key variables of physical fitness in previously untrained individuals.

## 5. Conclusions

Our study suggests that supplementation with 40 g of whey-based protein after a session of low-volume concurrent training can improve adaptations to low-volume concurrent training in previously untrained healthy individuals. Individuals combining low-volume endurance and resistance training in the same session may benefit from targeted protein supplementation, particularly to maximize leg muscle strength improvements.

## Figures and Tables

**Table 1:** Caloric and macronutrient contents of supplements (per serving size).

Variable	Protein Shake ^1,3^	Placebo Shake ^2,3^
Caloric value (kcal)	213	222
Protein (g)	40	5
Carbohydrates (g)	7.5	46
Fat (g)	2.6	2

^1^ Whey protein, ^2^ maltodextrin, ^3^ both shakes prepared with 150 mL low-fat milk.

**Table 2:** Dietary intake and physical activity during week 0 and week 8.

Outcome	PRO Group (N = 19)		PLA Group (N = 17)		Main Effect of Time (*p*-Value)	Group × Time Interaction (*p*-Value)
	Week 0	Week 8	Week 0	Week 8		
Nutrition ^1^						
Energy (kcal/day)	1987 ± 482	1965 ± 406	2052 ± 473	2000 ± 528	0.548	0.798
Protein (g/day)	81 ± 17	76 ± 11	79 ± 22	81 ± 28	0.656	0.237
Protein (g/kg/day)	1.2 ± 0.2	1.2 ± 0.3	1.1 ± 0.3	1.1 ± 0.3	0.786	0.511
Fat (g/day)	81 ± 28	78 ± 22	81 ± 23	80 ± 33	0.632	0.723
Fat (g/kg/day)	1.2 ± 0.4	1.2 ± 0.4	1.1 ± 0.4	1.1 ± 0.4	0.644	0.930
Carbohydrates (g/day)	207 ± 52	210 ± 78	216 ± 70	219 ± 63	0.745	0.995
Carbohydrates (g/kg/day)	3.2 ± 0.9	3.2 ± 1.0	3.0 ± 1.2	3.0 ± 1.0	0.929	0.990
Fiber (g/day)	21 ± 8	20 ± 8	22 ± 9	21 ± 9	0.527	0.830
Physical activity ^2^						
Light PA (h/week)	2.3 ± 1.4	2.4 ± 0.7	2.9 ± 0.7	2.9 ± 0.7	0.310	0.640
Moderate PA (h/week)	1.1 ± 0.2	1.1 ± 0.2	1.2 ± 0.5	1.4 ± 0.9	0.063	0.781
PAL	1.40 ± 0.02	1.41 ± 0.02	1.47 ± 0.01	1.47 ± 0.01	0.234	^3^

Data shown as means ± SD. Week 0 = 1 week before T-1, Week 8 = final week of intervention, PA = physical activity, PAL = estimated physical activity level. ^1^ Nutrition excluding the supplements, ^2^ PA excluding the study exercise program, ^3^ non-parametric testing.

**Table 3:** Anthropometric data at T-1 and T-2.

Outcome	PRO Group (N = 19)		PLA Group (N = 17)		Main Effect of Time (*p*-Value)	Group × Time Interaction (*p*-Value)
	T-1	T-2	T-1	T-2		
Body weight (kg)	65.9 ± 11.16	65.7 ± 11.6	75.9 ± 13.7	76.0 ± 12.9	0.936	0.530
Body mass index (kg/m^2^)	21.8 ± 2.2	21.8 ± 2.3	25.0 ± 4.3	25.0 ± 4.3	0.426	0.982
Fat mass (kg)	15.7 ± 4.3	15.4 ± 4.6	21.9 ± 10.2	22.0 ± 10.3	0.726	0.340
Fat mass (%)	24.0 ± 6.6	23.6 ± 6.6	28.3 ± 10.2	28.4 ± 10.4	0.535	0.238
Skeletal muscle mass (kg)	23.9 ± 6.1	24.0 ± 6.2	25.7 ± 5.5	25.9 ± 5.4	0.304	0.344
Total body water (L)	36.9 ± 7.6	36.0 ± 10.6	39.7 ± 7.2	39.8 ± 7.1	0.381	0.321
Waist circumference (cm)	74 ± 8	72 ± 8 ^a^	81 ± 7	80 ± 7	0.033	0.291

Data shown as means ± SD. T-1 = pre-intervention, T-2 = post-intervention. ^a^ *p* < 0.05: significant difference vs. T-1.

**Table 4:** Cardiorespiratory fitness data at T-1 and T-2.

Outcome	PRO Group (N = 19)		PLA Group (N = 17)		Main Effect of Time (*p*-Value)	Group × Time Interaction (*p*-Value)
	T-1	T-2	T-1	T-2		
VO_2max_ (mL/kg/min)	40.1 ± 6.3	42.8 ± 7.2 ^b^	36.8 ± 8.2	38.2 ± 8.1 ^a^	<0.001	0.210
VO_2max_ (L/min)	2.6 ± 0.8	2.9 ± 0.8 ^b^	2.8 ± 0.7	2.9 ± 0.7 ^a^	<0.001	0.166
W_max_ (W/kg)	3.2 ± 0.5	3.6 ± 0.5 ^c^	2.8 ± 0.6	3.0 ± 0.6 ^c^	<0.001	0.062
W_max_ (W)	213 ± 60	235 ± 64 ^c^	211 ± 43	228 ± 52 ^c^	<0.001	0.306
W_VT_ (W)	82 ± 36	90 ± 49	76 ± 12	78 ± 19	0.275	0.532

Data shown as means ± SD. VO_2max_ = maximal oxygen consumption, W_max_ = maximal power output, W_VT_ = power output achieved at ventilatory threshold. ^a^ *p* < 0.05, ^b^ *p* < 0.01, ^c^ *p* < 0.001: significant difference vs. T-1.

**Table 5:** One-repetition maximum strength data and Fit scores at T-1 and T-2.

Outcome	PRO Group (N = 19)		PLA Group (N = 17)		Main Effect of Time (*p*-Value)	Group × Time Interaction (*p*-Value)
	pre	post	pre	post		
Abdominals (kg)	28 ± 12	35 ± 14 ^c^	30 ± 11	34 ± 10	<0.001	0.050
Lower back(kg)	39 ± 13	47 ± 15 ^c^	49 ± 15	53 ± 14	0.001	0.276
Chest (kg)	34 ± 16	43 ± 16 ^c^	39 ± 19	47 ± 20 ^b^	<0.001	^1^
Upper back (kg)	47 ± 18	58 ± 22 ^c^	50 ± 20	57 ± 2 ^b^	<0.001	^1^
Legs (kg)	125 ± 42	152 ± 43 ^c^	132 ± 32	139 ± 35	<0.001	0.007
Fit score	47 ± 11	54 ± 13 ^c^	48 ± 9	52 ± 11 ^b^	<0.001	^1^

Data shown as means ± SD. ^b^ *p* < 0.01, ^c^ *p* < 0.001: significant difference vs. T-1. ^1^ Non-parametric testing.

## Data Availability

The datasets generated and analyzed during the current study are not publicly available, but are available from the corresponding author on reasonable request.
